# One Year of Yoga Training Alters Ghrelin Axis in Centrally Obese Adults With Metabolic Syndrome

**DOI:** 10.3389/fphys.2018.01321

**Published:** 2018-09-20

**Authors:** Angus P. Yu, Felix N. Ugwu, Bjorn T. Tam, Paul H. Lee, Christopher W. Lai, Cesar S. C. Wong, Wendy W. Lam, Sinead Sheridan, Parco M. Siu

**Affiliations:** ^1^School of Public Health, Li Ka Shing Faculty of Medicine, The University of Hong Kong, Pokfulam, Hong Kong; ^2^Department of Health Technology and Informatics, Faculty of Health and Social Sciences, The Hong Kong Polytechnic University, Hung Hom, Hong Kong; ^3^Department of Cell Biology and Physiology, The University of North Carolina at Chapel Hill, Chapel Hill, NC, United States; ^4^School of Nursing, Faculty of Health and Social Sciences, The Hong Kong Polytechnic University, Hung Hom, Hong Kong

**Keywords:** mind-body exercise, obesity, ghrelin, acylated ghrelin, unacylated ghrelin, obestatin, growth hormone, insulin

## Abstract

**Introduction:** Metabolic syndrome (MetS) is a multiplex cardiometabolic manifestation associated with type 2 diabetes mellitus and cardiovascular diseases. Yoga training has been shown to alleviate MetS. Recently, circulatory ghrelin profile was demonstrated to be associated with MetS. This study examined the effects of 1 year of yoga training on β-cell function and insulin resistance, and the involvement of metabolic peptides, including unacylated ghrelin (UnAG), acylated ghrelin (AG), obestatin, growth hormone (GH), and insulin, in the beneficial effects of yoga training in centrally obese adults with MetS.

**Methods:** This was a follow up study, in which data of risk factors of MetS, physical performance tests [resting heart rate (HR), chair stand test (CS), chair sit and reach test (CSR), back scratch test (BS), and single leg stand tests (SLS)] and serum samples of 79 centrally obese MetS subjects aged 58 ± 8 years (39 subjects received 1-year yoga training and 40 subjects received no training) were retrieved for analyses. β-cell function and insulin resistance were examined by Homeostasis Model Assessment (HOMA). Circulating levels of UnAG, AG, obestatin, GH, and insulin were determined by enzyme-linked immunosorbent assay using fasting serum samples. Generalized estimating equation analysis and Mann–Whitney *U*-test were used to detect statistically significant differences between groups.

**Results:** Waist circumference (WC) was significantly decreased after yoga intervention (control: +2%; yoga: -4%). Significant improvements in HR (control: +2%; yoga: -5%), CS (control: -1%; yoga: +24%), CSR left (control: worsen by 0.90 cm; yoga: improved by 4.21 cm), CSR right (control: worsen by 0.75 cm; yoga: improved by 4.28 cm), right side of BS (control: improved by 0.19 cm; yoga: improved by 4.31 cm), SLS left (control: -10%; yoga: +86%), and SLS right (control: -6%; yoga: +47%) were observed after 1-year yoga training. No significant difference was found between the two groups in insulin, HOMA indices, and disposition index. Yoga training significantly increased circulating GH (control: -3%; yoga: +22%), total circulating ghrelin (control: -26%; yoga: +13%), and UnAG (control: -27%; yoga: +14%), whereas decreased AG (control: -7%; yoga: -33%) and obestatin (control: +24%; yoga: -29%).

**Conclusion:** One-year of yoga training modulated total ghrelin, UnAG, AG, obestatin, and GH while exerting beneficial effects on physical functions and central obesity in adults with MetS. The beneficial effects of yoga may be associated with the alteration of ghrelin gene product and GH.

## Introduction

Metabolic syndrome (MetS) refers to a clustering of risk factors [central obesity, high blood pressure, high fasting blood glucose (GLU), high fasting triglycerides (TG), and low high-density lipoprotein-cholesterol (HDL-C)] that increase the risks of cardiovascular disease, type 2 diabetes and stroke. A recent systematic review from 15 countries in the Asia-Pacific region revealed that the prevalence of MetS is increasing, with approximately 20% of the Asian adult population being affected by the condition (Ranasinghe et al., 2017). We have recently reported that the prevalence of MetS is approximately 27% in Hong Kong (Siu et al., 2015).

While the majority of research to date has focused on more traditional risk factors associated with the development of MetS, recent investigations have led to identification of abnormal peptide profile of ghrelin gene products, growth hormone (GH), and insulin in individuals with MetS (Mora et al., 2014, 2015). Apart from the well-defined role of insulin in regulating blood GLU level, other aforementioned metabolic peptides were found to play important roles in regulating metabolic homeostasis. Different aspects of metabolism are complexly regulated by ghrelin gene products including unacylated ghrelin (UnAG), acylated ghrelin (AG), and obestatin (Gahete et al., 2014). Beyond its role as an orexigenic signal, ghrelin signaling has been recognized as a key regulator of obesity, insulin resistance and diabetes (Vijayakumar et al., 2011; Pradhan et al., 2013) and may involve in the pathological mechanisms of MetS (Langenberg et al., 2005). Individuals with MetS were observed to have lower total circulatory ghrelin (Langenberg et al., 2005). Previous study has demonstrated that individuals with either one of the MetS risk factors, except high TG, had lower ghrelin concentration in the blood (Ukkola et al., 2006). Ghrelin concentration in the blood was also positively correlated with HDL-C and negatively correlated with waist circumference (WC), systolic blood pressure (SBP), diastolic blood pressure (DBP), and fasting blood GLU (Ukkola et al., 2006). The circulatory total ghrelin and UnAG showed negative correlation to the number of risk factors of MetS while the relationship between AG and the number of risk factors of MetS remains controversial (Mora et al., 2015). The intrinsic interplay between AG and obestatin is implicated in several energy-related disorders such as obesity and MetS. AG has been demonstrated to be associated with insulin sensitivity (Gauna et al., 2004; Vestergaard et al., 2017) and blood pressure (Rodriguez et al., 2010). AG level has been shown to be higher in individuals with obesity and MetS (Rodriguez et al., 2010). Previous studies have demonstrated the involvement of obestatin in the obesity and diabetes, although the detailed mechanisms have not yet been discovered (Cowan et al., 2016). Children, adolescents and females with obesity have been demonstrated to have higher plasma obestatin levels but lower plasma total ghrelin levels (Vicennati et al., 2007; Razzaghy-Azar et al., 2016). Blood obestatin was positively correlated with total cholesterol and TG levels in obese women (Vicennati et al., 2007). Obestatin also contributes to the regulation of blood pressure and vascular function (Cowan et al., 2016). Study using animal model has demonstrated that obestatin stimulated the secretion of insulin and thus helped to regulate blood GLU level (Pradhan et al., 2017). Low GH level has been shown to be associated with obesity (Vijayakumar et al., 2011). Patients with GH deficiency have been demonstrated to have lower level of HDL-C (Carroll et al., 1998), and a higher prevalent rate of central obesity, hypertension, hypertriglyceridemia, and MetS (van der Klaauw et al., 2007). GH treatment was shown to reduce diastolic pressure, alleviate central obesity and improve lipid profile in centrally obese men (Johannsson et al., 1997). These data suggested that GH plays an important role in maintaining metabolic homeostasis.

Yoga has been recommended as an effective tool to improve metabolic disorders while it causes minimal or no adverse side effect (Sharma and Knowlden, 2012). Previous studies have demonstrated that yoga training reduced MetS z-score, central obesity, and SBP in individual with MetS (Lau et al., 2015; Siu et al., 2015). An 8-week Hatha yoga intervention was shown to reduce insulin level and HOMA-derived insulin resistance index in healthy subjects compared to their pre-intervention baseline values (Chen et al., 2016). Yoga exercise is professed to have health promoting effects by adherents but the overall effects of yoga on metabolic peptides remain unclear. The present study aimed to: (1) examine the effect of 1-year yoga training on β-cell function and insulin resistance by using HOMA model and (2) examine if the metabolic peptides, including UnAG, AG obestatin, GH, and insulin, associate with the beneficial effects of 1-year yoga training in centrally obese adults with MetS. We hypothesized that (1) yoga training alleviates insulin resistance and (2) the beneficial effects of yoga training are associated with the modulation of metabolic peptides.

## Materials and Methods

### Subject and Study Design

**FIGURE 1:**
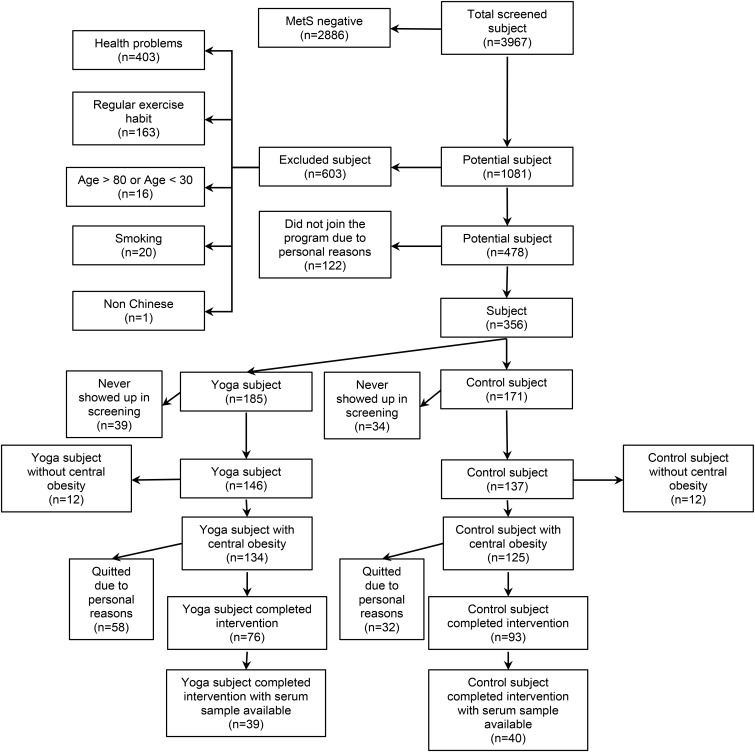
Subject flow diagram.

This follow up study examined centrally obese MetS middle-aged and older adults who participated in 1-year yoga intervention program or served as non-exercise control (Siu et al., 2015). In our previous study, we observed that 1-year yoga training reduced WC but not other individual MetS risk factors (Siu et al., 2015), suggesting that the effect of yoga might have been diluted by the vague combination of MetS risk factors with or without central obesity. To clarify the effect of 1-year yoga training on metabolic peptides including UnAG, AG, obestatin, GH, and insulin, the current investigation focused on MetS subjects with central obesity as a necessary risk factor among the MetS risk factors including elevated blood pressure, elevated TG, elevated fasting blood GLU and reduced HDL-C, as diagnosed by NCEP-ATP III prior to the commencement of the experiment. Our choice of MetS subjects with central obesity was also supported by findings that central obesity represents the most prevalent correlate of MetS (Després and Lemieux, 2006) and reports by International Diabetes Federation that central obesity is a necessary criterion to identify patients with MetS (Alberti et al., 2006). In the previous study, a total of 3,967 subjects were screened for MetS while 478 subjects were eligible (Siu et al., 2015). Among the 478 eligible subjects, 356 subjects joined the study and were randomly assigned to Control (*n* = 185) and Yoga (*n* = 171) groups (Siu et al., 2015). There were 283 subjects (Control: *n* = 137; Yoga: *n* = 146) participated in the assessment sections after randomization, while 259 of them had central obesity plus at least 2 of the 4 MetS risk factors as diagnosed by NCEP-ATP III (Control: *n* = 125; Yoga: *n* = 134) (Siu et al., 2015). Among these 259 central obese MetS subjects, there were 93 subjects in control group and 76 subjects in yoga group completed the study (dropout rate: control: 26%; yoga: 43%). Serum samples of 79 centrally obese MetS subjects (yoga, *n* = 39; control, *n* = 40; aged 58 ± 8 years; age range 41 to 77 years) were available for this current follow up study and were retrieved for analyses. The flow of subjects was summarized in **Figure 1**. Sera of the subjects before and after 1-year yoga intervention were analyzed by enzyme linked immunosorbent assay (ELISA) for UnAG, AG, obestatin, GH, and insulin. Written informed consent was obtained on a voluntary basis before the study began. All the experimental procedures were approved by The Hong Kong Polytechnic University (ethics approval number: HSEARS20160810001).

### Intervention

The subject randomization and intervention protocol adopted in this study has been previously described (Siu et al., 2015). Briefly, a computer program was employed to randomly assign all subjects into either a control or a yoga group. Monthly health status questionnaires and telephone calls were employed to monitor control subjects throughout the study whereas subjects in the yoga group were assigned to 1-year of yoga exercise training. The yoga training was conducted in small group (∼10 subjects in each group) led by certified yoga instructors and performed 3 times weekly for 1 year. Each session lasted approximately 1 h and consisted of 10-min of warm-up, 40-min of Hatha yoga practice, and 10-min of breathing exercise and relaxation cool-down. The postures in the routine training protocol during the 40 min of Hatha yoga practice includes sukhasana (easy pose), child pose, cat and cow stretch, adho mukha svanasana (downward dog), high lunge, uttanasana (standing forward bend), mountain pose, urdhva hastasana (upward salute), ukatassanna (chair pose), virabhadrasana (warrior pose), utthita parsva konasana (side angle pose), utthita trikonasana (extended triangle pose), vrksasana (tree pose), malasana (garland pose), eka pada rajakapotasana (one legged king pigeon pose), salambhasana (locust pose), dandasana (staff pose), baddha konasana (bound angle pose), agnistambhasana (fire log pose), gomukhasana (cow face pose), spinal twist, paripurna navasana (knees bend version of boat pose), setu bandhasana (bridge pose), supta padangusthasana (reclining big toe pose), ananda balsana (happy baby pose), centering (in cross-legged position), finger and toe weaving, virasana (hero pose), vajrasana (thunderbolt pose), tadasana (mountain pose), table pose (e.g., leg lifts), salabasana (locust pose), padangusthasana (big toe pose), sputa padangusthana (big toe lying down pose), utthita hasta padangustasana (extended big toe pose), sputa baddha konasana (lying down bound angle pose), eka pada bhekasana (1-leg frog pose), and shavasana (corpse pose). Subjects in the yoga group who failed to achieve 70% attendance to the training program were excluded in the study (Siu et al., 2015). All subjects were asked to adhere to their habitual daily dietary intakes and other physical activities throughout the experimental period.

### MetS Parameters

Measurements of blood pressure and WC were conducted before and after the 1 year experimental period by a trained research personnel. Fasting venous blood samples was obtained before and after the 1 year experimental period by certified phlebotomists for the measurements of fasting blood GLU, TG and HDL-C.

#### Blood Pressure

Systolic blood pressure (SBP), DBP, and resting heart rate (HR) were examined using an electronic blood pressure monitor (Accutorr Plus, Datascope). SBP and DBP were obtained over the brachial artery region on the right arm with the arm supported at heart level using appropriate sized cuff after 5-min seated rest. The average of two measurements taken with a 1-min interval was recorded for analysis (Siu et al., 2015).

#### Waist Circumference

Waist circumference (WC) was measured midway between the lowest rib and the superior border of the iliac crest using an inelastic measuring tape by snugging the tape horizontally around the abdomen passing across the navel on the bare skin during the end of normal expiration (Siu et al., 2015).

#### Fasting Blood Glucose, Triglycerides and High-Density Lipoprotein-Cholesterol

Venous blood samples were obtained by certified phlebotomists after a minimum of 10 h fast. Fasting plasma were harvested from the venous blood samples for biochemical measurements. Fasting blood GLU, TG, and HDL-C concentrations were measured by an accredited medical laboratory by commercial test kit methods using an automatic clinical chemistry analyzer (Architect CI8200, Abbott Diagnostics) (Siu et al., 2015).

### Physical Performance Tests

Resting HR was measured to indicate cardiac fitness status (Jensen et al., 2013), 30-s chair stand test (CS) was performed to assess the muscle strength of lower limb, chair sit and reach test (CSR), and back scratch test (BS) were used to assess the flexibility of subjects, and single leg stand test was employed to assess the balance of subjects. Physical performance was assessed before and after the 1 year experimental period by a trained research personnel.

#### Resting Heart Rate

Resting HR was examined by an electronic blood pressure monitor (Accutorr Plus, Datascope) during the assessment of blood pressure.

#### 30-S Chair Stand Test

The test began with a complete sitting position. During the test, subjects were asked to stand completely up and back completely down, and repeated this stand and sit cycle as many as possible within 30 s (Jones et al., 1999). The number of completed chair stand was recorded (Jones et al., 1999).

#### Chair Sit and Reach Test

Subjects were asked to sit at the edge of a chair with one leg extend forward with knee straight, heel on the floor and ankle bended as 90^o^, while the other foot remained flat on the floor (Jones et al., 1998). Subjects were instructed to place one hand over the other hand with the middle finger tips even, and tried to reach forward to the toe of the extended leg (Jones et al., 1998). The distance between the middle finger tips and the toe was recorded (Jones et al., 1998). If the finger tips did not reach to the toe, a negative value of distance was recorded and if the finger tips reach over the toe, a positive value of distance was recorded (Jones et al., 1998). The test was performed on both sides of legs.

#### Back Scratch Test

Subjects were in standing position and were asked to place one hand behind the head and back over the shoulder with palm facing the body, while placing the other arm behind the back with palm facing outward (Carvalho et al., 2009). Subjects were asked to make both hands reach as far as possible in attempted to touch or overlap the middle fingers (Carvalho et al., 2009). The distant between the two middle finger tips was recorded. If the middle fingers did not reach each other, a negative value of distant was recorded, and if the middle finger overlapped, a positive value of distant was recorded (Carvalho et al., 2009). The test was performed on both sides of arms.

#### Single Leg Stand Test

The time of subjects standing with one leg was measured. The timing was started when the subjects raised one of his/her foot off the ground (Macrae et al., 1992). Timing was stopped when their weight-bearing foot was displaced, their suspended foot touched the ground, suspended limb was used to support the weight-bearing limb, or reached the maximum balance time of 120 s (Macrae et al., 1992). The test was performed on both sides of legs. Subjects had three attempts of this test on each side. The best performed attempt was used for analysis.

### Peptide Determination, Beta Cell Function, and Insulin Resistance

Subjects’ serum samples and fasting blood GLU data before and after the 1-year experimental period were retrieved for the present study’s analyses. The sera were used for the measurement of UnAG, AG, obestatin, GH, and insulin. The measured insulin and the retrieved fasting blood GLU data were used for HOMA index calculations. All peptides were determined using commercially available ELISA kits by following the manufacturers’ instructions.

#### Beta Cell Function and Insulin Resistance

Homeostasis Model Assessment (HOMA) represents a computer-based model used to estimate beta-cell function and insulin resistance (Matthews et al., 1985). All HOMA indices were evaluated using fasting GLU and insulin. Since HOMA1, the first model, is limited in its functions, HOMA2, the current model, which allows for wider applications, even in hyperglycemic subjects was adopted in this study (Wallace et al., 2004). HOMA-%B2, HOMA-%S2, and HOMA-IR2 were calculated using the HOMA calculator^1^ whereas disposition index was calculated from multiplication of (HOMA-%S2)/100 by (HOMA-%B2)/100. HOMA-%B2, HOMA-%S2, and HOMA-IR2 represent β-cell function, sensitivity, and insulin resistance, respectively (Niemczyk et al., 2013).

#### UnAG and AG

Unacylated ghrelin and AG kits were purchased from BioVendor – Laboratorni medicina a.s., Karasek, Czechia (RA194063400R and RA194062400R, respectively). The assays were based on a double-antibody sandwich technique. Briefly, the wells of each human UnAG or AG antibody-coated microplate were rinsed before dispensing standards, dilution buffer, samples, quality controls and conjugate solution (blank) into appropriate wells. Plates were covered and incubated for 20 h at 4°C. Plate contents were discarded, rinsed adequately and blotted on paper towels to remove liquid traces. Substrate solution (Ellman’s reagent) was added to each well and the plate was read at 410 nm using a spectrophotometer. The plate was checked periodically every 30 min until maximum absorbance was attained. The results were calculated after the averages of the blank readings were subtracted from each well.

#### Obestatin

Human obestatin kits were purchased from RayBiotech, Norcross, GA, United States (EIA-OBS). Briefly, a biotinylated obestatin peptide was spiked into standards and samples. The samples were then added to appropriate wells where biotinylated obestatin competed with the endogenous obestatin for binding to the anti-obestatin antibody. The plates were incubated for 90 min at room temperature. The plates were washed prior to the introduction of horseradish peroxidase-streptavidin which catalyzed a color development reaction. Each plate was incubated at room temperature for 45 min, washed and blotted against a paper towel. The substrate reagent was added to each well, followed by 30 min of incubation in the dark. Stop solution was added and each plate was immediately read at 450 nm with a spectrophotometer.

#### Growth Hormone

Human GH kits were purchased from BioVendor – Laboratorni medicina a.s., Karasek, Czechia (RCD017R). The assay principle was based on a sandwich type assay. Briefly, calibrators, control and specimen samples were added to human antibody-GH-coated microplate wells. Conjugate solution was added to each well, followed by 60 min of incubation at room temperature. Wells were washed prior to introduction of substrate solution. Stop solution was added to each well after 15 min of incubation. Plates were read immediately using a spectrophotometer at 450 nm.

#### Insulin

Human insulin kits were purchased from BioVendor – Laboratorni medicina a.s., Karasek, Czechia (RA194062400R). The principle was based on a solid phase Enzyme Amplified Sensitivity Immunoassay performed on microplates. Briefly, monoclonal antibodies directed against distinct epitopes of insulin were introduced into the wells of each microplate. Calibrators, control and specimen samples were introduced to react with the capture monoclonal antibody (MAb 1) coated on the well and with a monoclonal antibody (MAb 2) labeled with horseradish peroxidase (HRP). After a 30-min incubation period allowing the formation of a sandwich: coated MAb 1-human insulin-MAb 2-HRP, each microplate was washed to remove unbound enzyme labeled antibody. Chromogenic solution was added to the wells, followed by 15 min of incubation. The reaction was terminated with the addition of stop solution and the microplate was then read at 450 nm (reference filter 630).

### Statistical Analysis

Values are expressed as mean ± SD. Normality of the baseline data were determined by Shapiro–Wilk Test. Baseline parameters of subjects in yoga and control groups including risk factors of MetS, physical performance tests, UnAG, AG, obestatin, GH, insulin, HOMA-%B2, HOMA-%S2, HOMA-IR2, and disposition index were analyzed by Mann–Whitney *U*-Test. The number of males and females in both groups were compared by Chi-Square Test. The main intervention effect, time effect and their interaction effect of group and time on UnAG, AG, obestatin, GH, insulin, HOMA-%B2, HOMA-%S2, HOMA-IR2, and disposition index were analyzed by generalized estimating equations (GEE). GEE was adopted as it is more robust on normality assumption. The comparisons of change in parameters between control and yoga groups were performed by Mann–Whitney *U*-Test. Statistical significance was accepted at *P* < 0.05. All statistical procedures were conducted using the Statistical Package for the Social Sciences (SPSS) version 24 for Windows.

## Results

The baseline characteristics of subjects in yoga and control groups including risk factors of MetS, physical performance tests, UnAG, AG, total ghrelin, obestatin, GH, insulin, HOMA-%B2, HOMA-%S2, HOMA-IR2, disposition index, International Physical Activity Questionnaire (IPAQ) activity and IPAQ sitting levels were summarized in **Table 1**. There was no significant difference in the baseline characteristics between yoga and control groups (**Table 1**).

**Table 1:** Baseline peptide levels and HOMA indices in MetS control and yoga subjects.

	Control group (*n* = 40)	Yoga group (*n* = 39)	*P*-value
Gender	8 male, 32 female	7 male, 32 female	0.955
Age	57.7 ± 8.6	58.8 ± 8.4	0.569
IPAQ activity (MET-min/week)	2824.9 ± 2315.9	4061.6 ± 4492.4	0.366
IPAQ sitting (min/week)	2301.0 ± 1511.5	2443.4 ± 1372.2	0.614
Metabolic parameters			
Waist circumference (cm)	89.25 ± 6.79	85.21 ± 7.72	0.687
Systolic blood pressure	134.03 ± 19.88	130.56 ± 19.54	0.659
Diastolic blood pressure	80.43 ± 10.41	80.72 ± 12.84	0.559
Fasting blood glucose	5.5 ± 0.66	5.80 ± 1.67	0.277
High-density lipoprotein-cholesterol	1.23 ± 0.30	1.24 ± 0.26	0.509
Triglyceride	2.15 ± 0.98	1.83 ± 0.76	0.336
Physical performance tests			
Resting heart rate	68.13 ± 7.53	70.95 ± 9.8	0.361
Chair stand test	15.30 ± 4.26	14.90 ± 4.67	0.577
Chair sit and reach test-left	–0.94 ± 6.69	+0.28 ± 7.46	0.572
Chair sit and reach test-right	–0.75 ± 6.48	+1.65 ± 7.99	0.243
Back stretch test-left	+5.83 ± 8.09	+4.41 ± 7.44	0.613
Back stretch test-right	+3.35 ± 6.33	+3.38 ± 7.65	0.814
Single leg stand test-left	71.68 ± 43.59	49.38 ± 39.95	0.063
Single leg stand test-left	64.48 ± 41.94	61.92 ± 35.78	0.840
Hormones			
Total ghrelin (pg⋅mL^-1^)	319.1 ± 199.9	265.3 ± 119.6	0.317
Unacylated ghrelin (pg⋅mL^-1^)	311.6 ± 201.2	258.7 ± 120.2	0.349
Acylated ghrelin (pg⋅mL^-1^)	7.5 ± 7.1	6.6 ± 4.7	0.627
Obestatin (pg⋅mL^-1^)	1.1 ± 0.7	1.1 ± 0.9	0.988
Growth hormone (ng⋅mL^-1^)	15.8 ± 4.7	18.5 ± 6.1	0.104
Insulin (pmol⋅L^-1^)	134.0 ± 32.7	132.6 ± 35.9	0.688
Beta cell function and insulin resistant			
HOMA2-%B	153.0 ± 42.1	143.7 ± 44.9	0.814
HOMA2-%S	42.1 ± 9.7	44.3 ± 18.5	0.483
HOMA2-IR	2.5 ± 0.6	2.5 ± 0.7	0.483
Disposition index	0.6 ± 0.1	0.6 ± 0.1	0.259


*Gender was analyzed by Chi-Square Test whereas other parameters were analyzed by Mann–Whitney U-Test. There was no significant difference in all metabolic parameters, physical performance tests, total ghrelin, unacylated ghrelin, acylated ghrelin, obestatin, growth hormone, insulin, HOMA2-%B, HOMA2-%S HOMA2-IR, and disposition index between control and yoga groups. MetS, metabolic syndrome; HOMA2-%B, homeostatic model assessment-β-cell function; HOMA2-%S, homeostatic model assessment-sensitivity; HOMA2-IR, homeostatic model assessment-insulin resistance; IPAQ, International Physical Activity Questionnaire. Significance level was set at P < 0.05.*

### Yoga Training Alleviated Central Obesity but Did Not Alter Other Risk Factors of MetS in Centrally Obese MetS Subjects

A significant interaction effect of group and time was observed in WC (*P* = 0.004). The changes in WC were significantly different between control and yoga groups (control: +2%; yoga: -4%, *P* = 0.001) (**Table 2**). No significant interaction effect of group and time, main effect of time, and main effect of group were observed in SBP, DBP, GLU, HDL, and TG (**Table 2**).

**Table 2:** Summary of Metabolic parameters and physical performance in central obese subjects with MetS in Control and Yoga groups.

	Control			Yoga			*P*-value				ICC
**Metabolic parameters**	**Pre**	**Post**	**Change**	**Pre**	**Post**	**Change**	**Time**	**Group**	**Interaction**	**Δ of parameter**	
WC (cm)	89.25 ± 6.79	90.84 ± 12.23	+1.59 ± 11.73	89.18 ± 6.85	85.21 ± 7.72	–3.97 ± 5.46	0.114	0.079	0.004	0.001	0.422
SBP (mmHg)	134.03 ± 19.88	128.65 ± 18.33	–5.38 ± 18.77	136.46 ± 17.85	130.56 ± 19.54	–5.90 ± 16.55	0.004	0.534	0.894	0.742	0.522
DBP (mmHg)	80.43 ± 10.41	78.85 ± 10.50	–1.58 ± 9.69	82.79 ± 9.39	80.72 ± 12.84	–2.08 ± 11.45	0.122	0.314	0.832	0.825	0.528
GLU (mmol/L)	5.5 ± 0.66	5.38 ± 0.95	–0.11 ± 0.63	5.82 ± 1.68	5.80 ± 1.67	–0.02 ± 0.65	0.348	0.196	0.517	0.188	0.884
HDL (mmol/L)	1.23 ± 0.30	1.26 ± 0.32	+0.03 ± 0.19	1.23 ± 0.27	1.24 ± 0.26	+0.01 ± 0.17	0.281	0.960	0.584	0.456	0.806
TG (mmol/L)	2.15 ± 0.98	2.08 ± 1.00	–0.07 ± 0.78	1.93 ± 0.83	1.83 ± 0.76	–0.10 ± 0.58	0.271	0.198	0.823	0.875	0.714
**Physical performance**								
HR (beat per min)	68.13 ± 7.53	69.55 ± 10.53	+1.43 ± 9.75	70.95 ± 9.8	67.13 ± 6.71	–3.82 ± 6.71	0.197	0.907	0.005	0.005	0.510
CS (times)	15.30 ± 4.26	15.20 ± 4.27	–0.10 ± 2.05	14.90 ± 4.67	18.49 ± 5.60	+3.59 ± 4.40	<0.001	0.141	<0.001	<0.001	0.681
CSR–left (cm)	+0.94 ± 6.69	–0.05 ± 7.82	–0.9 ± 3.19	–0.28 ± 7.46	+3.92 ± 8.29	+4.21 ± 4.98	0.001	0.396	<0.001	<0.001	0.797
CSR–right (cm)	+0.75 ± 6.48	+0.00 ± 7.93	–0.75 ± 3.33	–1.65 ± 7.99	+2.63 ± 7.34	+4.28 ± 5.62	0.001	0.943	<0.001	<0.001	0.758
BS-left (cm)	–5.832± 8.09	–6.00 ± 7.11	–0.17 ± 5.55	–4.41 ± 7.44	–3.62 ± 6.85	+0.79 ± 6.27	0.636	0.206	0.463	0.177	0.319
BS-right (cm)	–3.35 ± 6.33	–3.16 ± 6.59	+0.19 ± 4.99	–3.38 ± 7.65	+0.93 ± 6.58	+4.31 ± 6.55	0.001	0.138	0.001	0.001	0.606
SLS-left (s)	71.68 ± 43.59	64.68 ± 43.00	–7.00 ± 28.80	49.38 ± 39.95	91.95 ± 34.71	+42.56 ± 58.85	0.001	0.735	<0.001	<0.001	0.233
SLS-right (s)	64.48 ± 41.94	61.18 ± 40.93	–3.63 ± 18.93	61.92 ± 35.78	91.00 ± 32.17	+29.08 ± 35.24	<0.001	0.084	<0.001	<0.001	0.657


*The main effect of group, main effect of time and interaction effect of group and time on metabolic parameters and physical performance were analyzed by generalized estimating equation (GEE). Significance level was set at P < 0.05. ICC, intra-class correlation coefficient; Δ of parameter, change in parameter; WC, waist circumference; SBP, systolic blood pressure; DBP, diastolic blood pressure; GLU, fasting blood glucose; HDL, high-density lipoprotein-cholesterol; TG, triglycerides; HR, resting heart rate; CS, 30-s chair stand test; CSR, chair sit and reach test; BS, back scratch test; SLS, single leg stand test.*

### Yoga Training Improved Physical Performance in Centrally Obese MetS Subjects

A significant interaction effect of group and time was observed in HR (*P* = 0.005).The decrease in HR in yoga group was significantly larger than that of control group (control: +2%; yoga: -5%, *P* = 0.005) (**Table 2**). A significant interaction effect of group and time was observed in the 30-s CS (*P* < 0.001). The improvement in the performance of 30-s CS of yoga group was significantly larger than that of control group (control: -1%; yoga: +24%, *P* = 0.005) (**Table 2**). Significant interaction effects of group and time were observed in CSR-left (*P* < 0.001) and CSR-right (*P* < 0.001) (**Table 2**). The improvements in CSR of both sides in yoga group were significantly larger than that of control group (left side: control: worsen by 0.90 cm; yoga: improved by 4.21 cm, *P* < 0.001; right side: control: worsen by 0.75 cm; yoga: improved by 4.28 cm, *P* < 0.001) (**Table 2**). A significant interaction effect of group and time was observed in the right side of the BS (*P* = 0.001) (**Table 2**). The improvement in BS-right of yoga group was significantly larger than that of control group (control: improved by 0.19 cm; yoga: improved by 4.31 cm, *P* = 0.001) (**Table 2**). No significant change was observed in the left side of the BS (**Table 2**). Similar pattern was observed in the single leg stand test. Significant interaction effects of group and time were observed in both single leg stand test-left (*P* < 0.001) and single leg stand test-right (*P* < 0.001) (**Table 2**). The improvements in single leg stand test of both sides in yoga group were significantly larger than that of control group (left side: control: -10%; yoga: +86%, *P* < 0.001; right side: control: -6%; yoga: +47%, *P* < 0.001) (**Table 2**).

### Yoga Training Had No Effect on β-Cell Function and Insulin Resistance, but a Tended Effect on Improving Insulin Sensitivity

Neither interaction effect of group and time, main effect of time nor main effect of group was observed in HOMA-%B2, HOMA-%S2, HOMA-IR2, and disposition index (**Table 3**). Notably, a tended significant group effect was observed in HOMA-%S2. The HOMA-%S2 of yoga group was increased by 23% while only 2% increase was observed in control group. However, the changes in HOMA-%S2 were not significantly different between yoga and control group (*P* = 0.537; **Table 3**). The change in HOMA-%B2, HOMA-IR2, and disposition index before and after intervention were not differed between control and yoga groups (**Table 3**).

**Table 3:** Summary of ghrelin gene products, growth hormone, insulin and HOMA indices in central obese subjects with MetS in Control and Yoga groups.

	Control			Yoga			*P*-value				ICC
**Beta cell function and insulin resistance**	**Pre**	**Post**	**Change**	**Pre**	**Post**	**Change**	**Time**	**Group**	**Interaction**	**Δ of parameter**	
HOMA2-%B	153.03 ± 42.10	164.68 ± 59.48	+11.64 ± 59.91	143.68 ± 44.95	144.27 ± 61.66	+0.59 ± 52.84	0.329	0.133	0.378	0.303	0.433
HOMA2-%S	42.13 ± 9.68	42.91 ± 12.50	+0.78 ± 14.15	44.30 ± 18.45	54.56 ± 40.02	+10.26 ± 33.87	0.057	0.061	0.101	0.537	0.384
HOMA2-IR	2.50 ± 0.59	2.54 ± 0.79	+0.04 ± 0.90	2,51 ± 0.67	2.53 ± 1.11	+0.02 ± 1.08	0.807	0.992	0.926	0.875	0.256
Disposition index	0.62 ± 0.15	0.67 ± 0.17	+0.04 ± 0.11	0.59 ± 0.14	0.63 ± 0.22	+0.04 ± 0.19	0.009	0.306	0.985	0.550	0.611
Hormones					
Total ghrelin (pg/ml)	319.06 ± 199.91	235.67 ± 105.48	–83.39 ± 222.35	265.27 ± 119.58	299.88 ± 209.48	+34.61 ± 242.83	0.346	0.842	0.023	0.055	0.023
Acylated ghrelin (pg/ml)	7.48 ± 7.05	6.93 ± 5.71	–0.55 ± 9.39	6.59 ± 4.74	4.43 ± 2.43	–2.16 ± 5.61	0.113	0.042	0.348	0.046	0.007
Unacylated Ghrelin (pg/ml)	311.57 ± 201.21	228.74 ± 105.09	–82.83 ± 223.02	258.68 ± 120.16	295.46 ± 209.82	+36.77 ± 243.88	0.375	0.792	0.021	0.051	0.025
Obestatin (pg/ml)	1.07 ± 0.72	1.33 ± 0.98	+0.26 ± 1.04	1.10 ± 0.94	0.78 ± 0.71	–0.32 ± 1.26	0.827	0.048	0.026	0.102	0.031
Growth hormone (ng/ml)	15.78 ± 4.73	15.32 ± 45.79	–0.46 ± 5.22	18.53 ± 6.14	22.53 ± 9.19	+4.00 ± 8.92	0.030	<0.001	0.006	0.043	0.459
Insulin (pg/ml)	133.98 ± 32.68	137.32 ± 45.79	+3.34 ± 52.00	132.62 ± 35.89	134.25 ± 59.88	+1.63 ± 59.24	0.688	0.777	0.891	0.930	0.227


*The main effect of group, main effect of time and interaction effect of group and time on hormones, beta cell function and insulin resistant were analyzed by generalized estimating equation (GEE). Statistical significance was accepted at P < 0.05. Significance level was set at P < 0.05. ICC, intra-class correlation coefficient; Δ of parameter, change in parameter; HOMA2-%B, homeostatic model assessment-β-cell function; HOMA2-%S, homeostatic model assessment-sensitivity; HOMA2-IR, homeostatic model assessment-insulin resistance.*

### Yoga Training Decreased AG and Obestatin and Increased UnAG and Total Ghrelin

There was a significant interaction effect of group and time on total ghrelin (*P* = 0.023) (**Table 3**). A tended significant difference was observed in the change in total ghrelin between the two groups (control: -26%; yoga: +13%, *P* = 0.055) (**Table 3**). There was no significant interaction effect of group and time on AG (**Table 3**). However, there was a significant main effect of group on AG (*P* = 0.042) (**Table 3**). The decrease in AG of yoga group was significantly larger than that of control group (control: -7%; yoga: -33%, *P* = 0.046) (**Table 3**). There was a significant interaction effect of group and time on UnAG (*P* = 0.021) (**Table 3**). A tended significant difference was observed in the changes in total ghrelin between the two groups (control: -27%; yoga: +14%, *P* = 0.051) (**Table 3**).There was a significant interaction effect of group and time on obestatin (*P* = 0.026) (**Table 3**). The obestatin level in control group was increased by 24%, while the obestatin level in yoga group was decreased by 29%, however, the changes in obestatin level between yoga group and control group did not reach to a statistical significance (*P* = 0.102).

### Yoga Training Increased GH but Not Insulin

There was a significant interaction of group and time on GH (*P* = 0.006) (**Table 3**). The change in GH in yoga and control groups were significantly different (control: -3%; yoga: +22%, *P* = 0.043) (**Table 3**). No significant interaction effect of group and time was found on insulin (**Table 3**). Neither the time nor group effect on insulin level was found (**Table 3**).

## Discussion

On top of demonstrating the beneficial effects of yoga intervention on alleviating central obesity and improving physical performance, we investigated the effects of 1-year yoga training on β-cell function, insulin resistance, and metabolism-related peptides, including GH, insulin, and ghrelin gene products namely UnAG, AG, total ghrelin, and obestatin, in MetS adults with central obesity. Our results demonstrated that yoga intervention did not improve β-cell function and insulin resistance in centrally obese MetS adults, except a tended improvement in insulin sensitivity indicated by HOMA2-%S. One-year yoga training increased the fasting circulatory level of total ghrelin, UnAG, and GH, whereas decreased the AG and obestatin.

Previously study showed that yoga intervention reduced insulin level and HOMA-derived insulin resistance index in healthy subjects compared to their pre-intervention baseline values (Chen et al., 2016). It was also demonstrated that yoga exercise increased the sensitivity of the pancreas islets to GLU signal in healthy subjects who had performed different combinations of yoga postures for five consecutive days (Manjunatha et al., 2005). However, in our study, insulin level, HOMA2-%B, HOMA2-IR, and disposition index were not significantly altered in yoga and control groups while only a tended increase in HOMA2-%S was observed in yoga group relative to control group. The absence of changes in HOMA2 indices were in line with the similar GLU metabolism observed in intervention and control groups indicated by the fairly constant disposition index (Cobelli et al., 2007). This data suggested that the effects of yoga on manipulating insulin level, improving beta-cell function and insulin resistant may be diminished in centrally obese MetS individuals.

We have previously reported that 1-year yoga intervention decreased WC and tended to decrease SBP in MetS individuals (Siu et al., 2015). In this study, it is observed that 1-year yoga intervention reduced WC in centrally obese MetS individuals. These data suggested that yoga intervention alleviates MetS. As total ghrelin is negatively correlated with WC and the number of metabolic risk factors of MetS (Ukkola et al., 2006), our observed significant increase in total ghrelin after 1-year yoga intervention was in line with the alleviation of MetS. Consistent with our results of decreased WC and increased total ghrelin after the yoga intervention, a study in 2005 has demonstrated that total ghrelin was increased after 1-year of aerobic exercise in overweight postmenopausal women accompanied with weight loss and decrease in WC (Foster-Schubert et al., 2005).

Most of the previous studies that solely examined total ghrelin may not be able to identify the independent effects of the distinct forms of ghrelin. A key strength of the current study is the evaluation of both forms of ghrelin – UnAG and AG. It has become increasingly recognized that UnAG plays crucial roles in metabolism. Fasting UnAG has been demonstrated to be significantly higher in normal healthy subjects when compared to individuals with MetS risk factor such as central obesity (Dardzinska et al., 2014). In a 2-week clinical trial, single daily injection of AZP-531, an UnAG agonist, decreased body weight from baseline in obese subjects (Allas et al., 2016). In this current study, the increase in UnAG in intervention group after 1-year of yoga training might indicate the potential of UnAG in restoring metabolic homeostasis in MetS subjects. Indeed, it has been demonstrated that UnAG is related to changes in body composition and fat distribution after a long-term exercise intervention (Cederberg et al., 2011). Previous studies showed that UnAG, but not AG was increased after aerobic exercise intervention. It has been demonstrated in obese women that UnAG, but not AG, increased with weight loss after 8-week of mild running exercise (Mirzaei et al., 2009). Similarly, 12-week of combined walking and rubber band exercises was shown to increase UnAG by over 100%, whereas AG concentration was unaltered in overweight boys aged 11 years in comparison to control group (Kim et al., 2008). These data suggested that studying both forms of ghrelin, other than total ghrelin alone, could facilitate the study of ghrelin axis in regulating energy balance and weight. Besides decrease in total ghrelin and UnAG, increase in AG/UnAG and AG level was also observed in obese individuals (Barazzoni et al., 2007). Contrary to the studies reporting no change in AG after aerobic exercises, the present study found a decrease in AG after the yoga intervention. Yoga, as a mind-body exercise, may have different effects on modulating the metabolic peptides compared with traditional aerobic exercise. As AG level is positively associated with SBP (Rodriguez et al., 2010) and negatively associated with insulin sensitivity (Gauna et al., 2004; Barazzoni et al., 2007; Vestergaard et al., 2017), the decreased AG level after yoga intervention may imply a potential beneficial effects on improving blood pressure and insulin sensitivity. Indeed, a systematic review concluded that yoga training can serve as an alternative treatment of hypertension (Park and Han, 2017) and diabetes (Mondal et al., 2018). The decreased AG may partly contribute to the tended group effect of the higher insulin sensitivity observed in yoga group compared with control group after 1-year experimental period. The absence of significant decrease in blood pressure and fasting blood GLU level after yoga intervention may due to the fact that some subjects were having normal blood pressure and fasting blood GLU as both high blood pressure and impaired GLU were not necessary inclusion criteria in the present study. Further investigation is needed to examine if the beneficial effects of yoga on high blood pressure and fasting blood GLU are associated with the level of AG in the populations with hypertension or high blood GLU. Obestatin is associated with lipid profile (Vicennati et al., 2007), blood pressure (Cowan et al., 2016), and blood GLU (Pradhan et al., 2017). Previous studies have suggested that obestatin plays an important role in obesity and diabetes, however, inconsistency was observed in the data of the relationship between obestatin and obesity/diabetes (Cowan et al., 2016). Our data agreed with previous studies that obestatin has a positive relationship with obesity and MetS (Vicennati et al., 2007; Mora et al., 2013). Detailed mechanisms of how obestatin contributes to the modulation of metabolic parameters warrants further investigation.

The actions of GH are diverse and complex, affecting virtually all tissues of the body. Lower GH level has been demonstrated to increase obesity (Vijayakumar et al., 2011), while GH treatment can improve DBP, lipid profile, and alleviate central obesity (Johannsson et al., 1997). Exercise has been demonstrated to be a potent stimulus for GH secretion, which leads to lipolysis (Sonksen, 2001). The increase in GH level after yoga intervention may favor lipolysis and thus alleviates central obesity. As the relationship between GH and blood pressure has previously been demonstrated (Johannsson et al., 1997), the increase in GH level may contribute to the beneficial effects of yoga on blood pressure. Further investigation is needed to examine if the yoga-induced increase in GH is associated with improvement of blood pressure in hypertensive patients.

Notably, dietary habit plays an important role in metabolic homeostasis. Subjects in this study were reminded not to change their dietary habits during the experimental period during the monthly phone calls and the yoga lessons. We have obtained dietary data in a random sub-sample. It has been reported in the original study that the dietary habit of the random sub-sample was not altered throughout the 1 year experimental period (Siu et al., 2015).

To our best understanding, this is the first study reporting the effects of yoga training on altering the circulating ghrelin gene products. Our findings that 1-year yoga training increased the circulating level of GH, total ghrelin and UnAG but decreased AG and obestatin substantiate the previous findings that implicated the role of ghrelin gene products in the progression of metabolic disorders. Although the exact mechanisms by which yoga exercise alters the concentrations of GH and ghrelin gene products remains unclear, our study affirms the theory that disproportion of circulatory ghrelin gene products might be related to the pathophysiology of MetS and therapies aimed at normalizing MetS risk factors might function in part by restoring the levels of ghrelin gene products. Further studies are warranted to delineate the mechanisms by which yoga exercise alters the production, secretion and/or turnover of GH and ghrelin gene products.

This study has several limitations. Subjects in this study had central obesity plus at least 2 of the 4 MetS risk factors rather than having identical profile of risk factors, which means that parts of the subjects had normal blood pressure, triglycerides, and HDL-C. The data from subjects with normal blood pressure, triglycerides and HDL-C may have diluted the effects of yoga intervention on improving those metabolic parameters. This study included both female and male subjects in the two groups. Gender effect has previously been reported in the metabolic peptides and metabolic responses (Abu-Farha et al., 2014). The gender difference may also have diluted the effects of yoga on the metabolic peptides and metabolic responses. Nonetheless, the conclusion of this study should be minimally affected by the gender effect due to the very similar gender ratios for the two groups. We tried to further address this issue by performing the analysis with only female or male samples (data not shown). All the results were reproducible in the female samples but significant changes were only observed in WC, CS, and CSR and single leg stand test in the male subjects. The different results obtained from female and male samples might be due to the gender effect on the metabolic peptides and metabolic responses. However, the effect of gender remains inconclusive due to the small sample size of males in each group (8 men in control group and 7 men in yoga group). We also noticed that the intra-class coefficient was low in ghrelin gene produces, which may be due to the wide range of the level of circulating ghrelin gene product among subjects.

## Conclusion

In conclusion, 1-year yoga intervention modulated the circulating level of ghrelin gene products and GH, while providing beneficial effects on physical performance and central obesity in adults with MetS. The beneficial effects of yoga might be mediated by ghrelin gene products and GH. Further investigations are needed to identify the roles of ghrelin gene products and GH on the beneficial effects of yoga.

## Author Contributions

AY and FU contributed to the study design, data collection, data analysis, analysis of the paper, and writing of the paper. BT contributed to data collection. PL contributed to data analysis and analysis of the paper. CL, CW, WL, and SS contributed to analysis of the paper. PS contributed to the study design, analysis of the paper, and writing of the paper.

## Conflict of Interest Statement

The authors declare that the research was conducted in the absence of any commercial or financial relationships that could be construed as a potential conflict of interest.

## Footnotes

1 https://www.dtu.ox.ac.uk/homacalculator/
